# Effect of functional group ratio in PEBAX copolymer on propylene/propane separation for facilitated olefin transport membranes

**DOI:** 10.1038/s41598-019-47996-7

**Published:** 2019-08-07

**Authors:** Kyoung Won Jung, Sang Wook Kang

**Affiliations:** 1 Department of Chemistry, Sangmyung University, Seoul, 03016 Republic of Korea; 2 Department of Chemistry and Energy Engineering, Sangmyung University, Seoul, 03016 Republic of Korea

**Keywords:** Pollution remediation, Polymers

## Abstract

PEBAX-5513/AgBF_4_/Al(NO_3_)_3_ membranes were fabricated for mixed olefin/paraffin separation. In order to improve the selectivity of the membranes utilizing PEBAX-1657, PEBAX-5513, which increased the ratio of amide groups from 40% to 60% in the copolymer, was used. The selectivity and permeance of the membranes were 7.7 and 11.1 GPU, respectively. Furthermore, the PEBAX–5513/AgBF_4_/Al(NO_3_)_3_ membranes had long-term stability because of Al(NO_3_) to have the stabilizing effect on Ag^+^ ions acting as an olefin carrier. Unexpectedly, the performance of the membrane selectivity was not improved, and the permeance became rather lower. Generally, when Ag^+^ ions was added to the polymer containing amide groups, the selectivity increased with the content of the amide groups. However, Al(NO_3_)_3_ was added for the stability of Ag^+^ ions and there was no increase in selectivity. Since the ratio of amide was high, Ag^+^ ions were favorably in coordination with the oxygen of the carbonyl group, but the NO_3_^−^ ions in Al(NO_3_)_3_ had the enhanced interaction with Ag^+^ ions as obstacles for olefin complexation. Therefore, the composition ratio of amide/ether in the polymer matrix was negligible for olefin separation.

## Introduction

Recently, the separation of propylene/propane mixtures has become important in industrial processes^1^. Olefins and paraffins have similar chemical characteristics; therefore, the fuel efficiency has been found to be very low when fuel is used without separating them^2^. Hence, the techniques of their separation have been in the spotlight^3^. However, conventional separation methods showed problems such as high cost and low efficiency^4^; hence, recently, separation methods using membranes have attracted much attention^5^. Especially, polymeric membranes made it possible for energy, process cost and process to be efficient^6–8^.

The facilitated transport of polymer membranes using Ag^+^ ions from precursors such as AgBF_4_, AgCF_3_SO_3_, and AgNO_3_ showed prominent achievement for olefin/paraffin separation^9–12^. It was proved that the permeance and selectivity of the membrane increased upon the addition of the Ag salts. Furthermore, many polymers such as poly(2-ethyl-2-oxazoline) (POZ), polyvinylpyrrolidone (PVP)^13^, and poly(vinyl alcohol) (PVA)^14^ were used as the matrices of the membrane to improve the membrane properties.

However, Ag^+^ ions were easily reduced over time to form particles, and these particles have the disadvantage of interfering with the permeation of gases. Our group reported a membrane with Al(NO_3_)_3_ added to delay the reduction of the Ag^+^ ions, and to maintain the long-term durability of the membrane^15^. The membrane properties of the POZ/AgBF_4_/Al(NO_3_)_3_ membrane lasted for more than 14 days, and the white color of the membranes remained unchanged for 3 months due to the reduction of Ag^+^ ions^15^. These phenomena were generated from that the Al^3+^ ions in Al(NO_3_)_3_ had a favorable electrostatic interaction with the BF_4_^−^ ions in AgBF_4._ Thus, the interaction between BF_4_^−^ ions and its counter ion, Ag^+^ ions, was relatively weaker and the Ag^+^ ions as an olefin carrier was freed without reduction^16^. As a result, the POZ/AgBF_4_/Al(NO_3_)_3_ membrane had long-term stability. However, unfortunately, it had a relatively low permeance of 4.8 GPU^15^.

To improve the permeance, we used a highly permeable polymer, PEBAX-1657, as the membrane matrix, in an earlier study^6^. Using the complex with PEBAX-1657 as the matrix, we achieved an enhanced performance of 22.5 GPU with 8.8 selectivity^6^.

Poly(ether-block-amide) resin, a typical thermoplastic elastomer, was widely known as PEBAX. PEBAX was consisted of a linear chain combination of polyamide, a hard segment responsible for mechanical strength, and polyehter, a soft segment responsible for high permeability due to high chain mobility of the ether linkage^17^.

In case of PEBAX-1657, 60% of the permeable ether group and 40% of the hard amide group were able to obtain a high permeance, but the selectivity tended to be relatively low^6^.

In this study, to increase the selectivity, PEBAX-5513, which increased the ratio of the amide groups capable of showing high selectivity, was selected as the polymer matrix.

## Results and Discussion

### SEM images analysis

**Figure 1:**
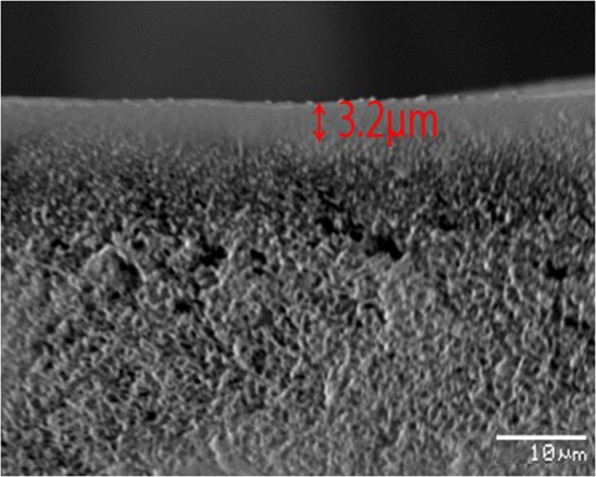
Scanning electron microscopy (SEM) image of the membrane: polysulfone support membrane coated with PEBAX-5513/AgBF_4_/Al(NO_3_)_3._

SEM images were obtained to confirm that the selective layer was uniformly distributed. Figure 1 shows the cross-section of the selective layer of PEBAX-5513/AgBF_4_/Al(NO_3_)_3_ coated on the porous polysulfone. The image shows that the selective layer of PEBAX-5513/AgBF_4_/Al(NO_3_)_3_ was uniformly coated and the thickness was 3.2 µm.

### Separation performance

**Figure 2:**
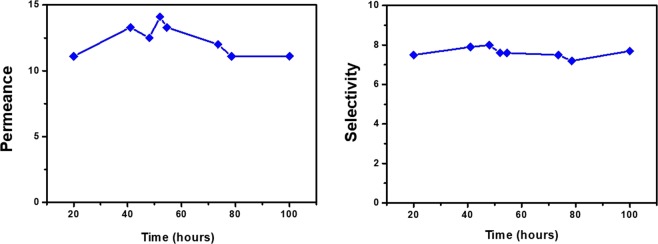
Separation performance of PEBAX-5513/AgBF_4_/Al(NO_3_)_3_ membrane: (**a**) mixed gas permeance and (**b**) selectivity for propylene/propane 50:50 (v/v) mixture.

Figure 2 shows the permeance and selectivity of the PEBAX-5513/AgBF_4_/Al(NO_3_)_3_ membrane for a propylene/propane mixture. The permeance and selectivity of the membrane were about 11.1 GPU and 7.7, respectively. Furthermore, Ag^+^ ions could act as olefin carriers, owing to Al(NO_3_)_3_, a reduction inhibitor. Thus, the long-term stability of the membrane was confirmed over 100 h. In another earlier study, a high selectivity was observed due to the coordination of the carbonyl oxygens of the amide group with the Ag^+^ ions^18–22^. However, similar results were not observed in this study. The reason for these phenomena is discussed in the next section.

### FT-IR analysis

**Figure 3:**
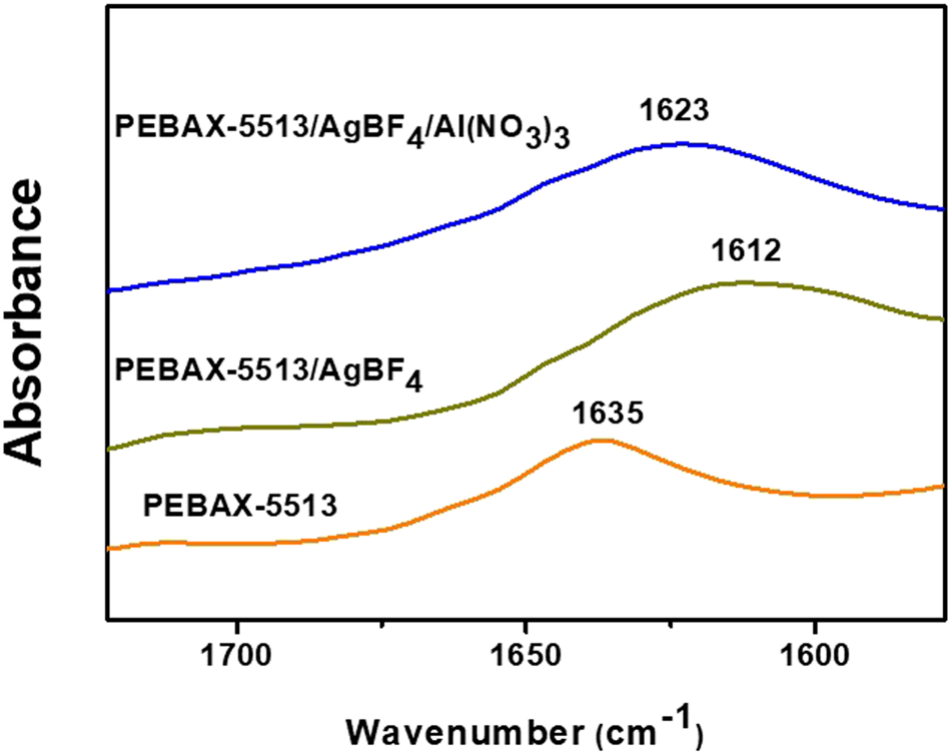
FT-IR spectra of neat PEBAX-5513, PEBAX-5513/AgBF_4_, and PEBAX-5513/AgBF_4_/Al(NO_3_)_3._

Figure 3 shows the FT-IR spectrum of the neat PEBAX-5513, PEBAX-5513/AgBF_4_, and PEBAX-5513/AgBF_4_/Al(NO_3_)_3_. For the neat PEBAX-5513 spectrum, the peak of C=O bond was observed at 1635 cm^−1^, but the peak moved to 1612 cm^−1^ when AgBF_4_ was added. This was because the intensity of the C=O bond peak was weakened as the Ag^+^ ions of AgBF_4_ react with the oxygen atom of the C=O bond in the amide group of the polymer. Thereafter, when Al(NO_3_)_3_ was added to PEBAX-5513/AgBF_4_, the peak of C=O bond shifted to 1623 cm^−1^. This was due to the NO_3_^−^ ions interacting with the Ag^+^ ions bound to the oxygen atom of the C=O. Thus, the intensity of the C=O bond peak was restored, and the C=O bond peak shifted to a higher. For PEBAX-1657, when AgBF_4_ was added, the wavenumber of the C=O bond peak slightly decreased from 1637 to 1620 cm^−1^ ^6^; however, for PEBAX-5513, it decreased significantly from 1635 to 1612 cm^−1^. This was because PEBAX-5513 had a higher proportion of amide groups than that of PEBAX-1657, since Ag^+^ more favorably coordinates to the amide group than the ether group. However, when Al(NO_3_)_3_ was added, the recovered wavenumber of the C=O bond peak was similar for both PEBAX-5513 and PEBAX-1657. Hence, it was thought that the degree of coordination of the NO_3_^−^ ions of Al(NO_3_)_3_ with Ag^+^ ions was similar.

### TGA analysis

**Figure 4:**
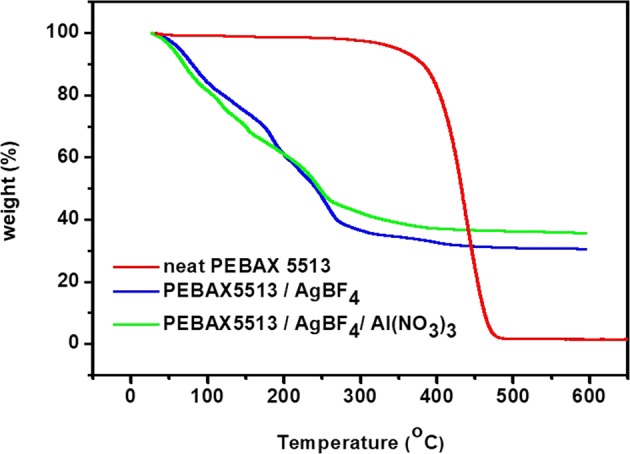
TGA graph of neat PEBAX-5513, PEBAX-5513/AgBF_4_, and PEBAX-5513/AgBF_4_/Al(NO_3_)_3._

To investigate the thermal stability of neat PEBAX-5513, PEBAX-5513/AgBF_4_, and PEBAX-5513/AgBF_4_/Al(NO_3_)_3_, TGA analysis was performed from 0 to 600 °C as shown in Fig. 4. In PEBAX-5513/AgBF_4_, although the Ag^+^ ions formed a bridge between the polymer chains, the Ag^+^ ions coordinated to the amide group weakened the existing intermolecular attractions in the polymer. The enhanced free volume of the polymer caused plasticization. As a result, the thermal stability seemed to decrease. However, when Al(NO_3_)_3_ was added, the results were different from those for PEBAX-1657. The thermal stability of PEBAX-1657 slightly increased^6^, since the NO_3_^−^ ions of Al(NO_3_)_3_ formed a new bond with the Ag^+^ ions coordinated to the C=O bond, weakening the bonds between oxygen and Ag^+^ ions. On the contrary, when Al(NO_3_)_3_ was added to PEBAX-5513/AgBF_4_, even the crosslinking formed between the Ag^+^ ions and polymer chain collapsed, and the thermal stability was expected to be lower.

### Raman analysis

**Figure 5:**
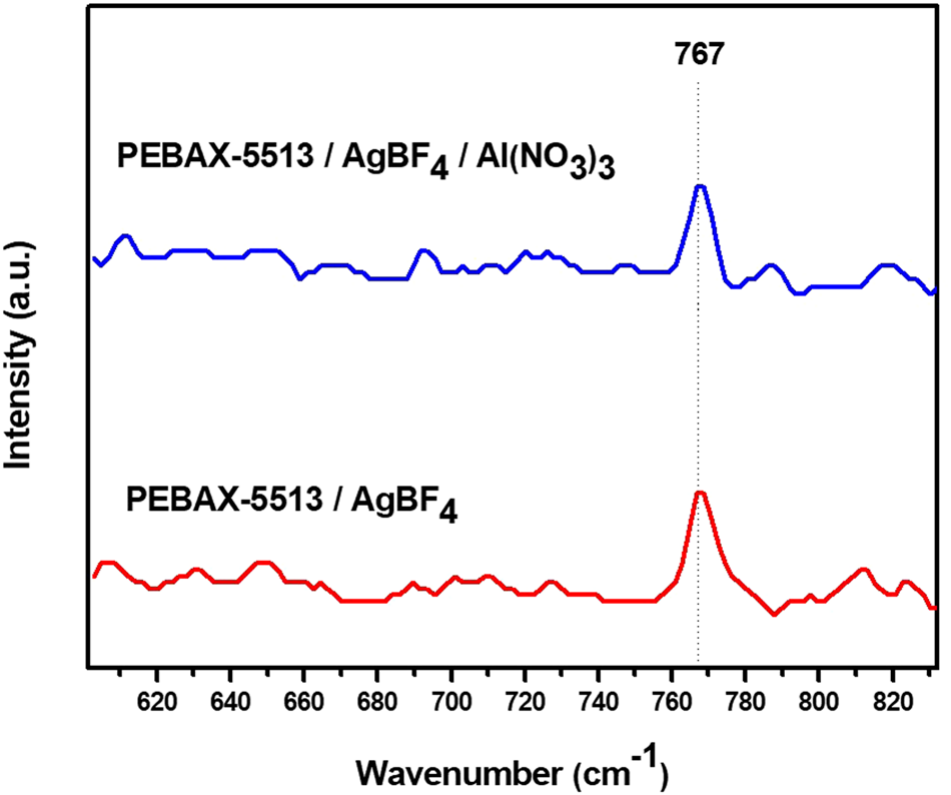
Raman spectra of BF_4_^−^ in PEBAX-5513/AgBF_4_ and PEBAX-5513/AgBF_4_/Al(NO_3_)_3._

**Figure 6:**
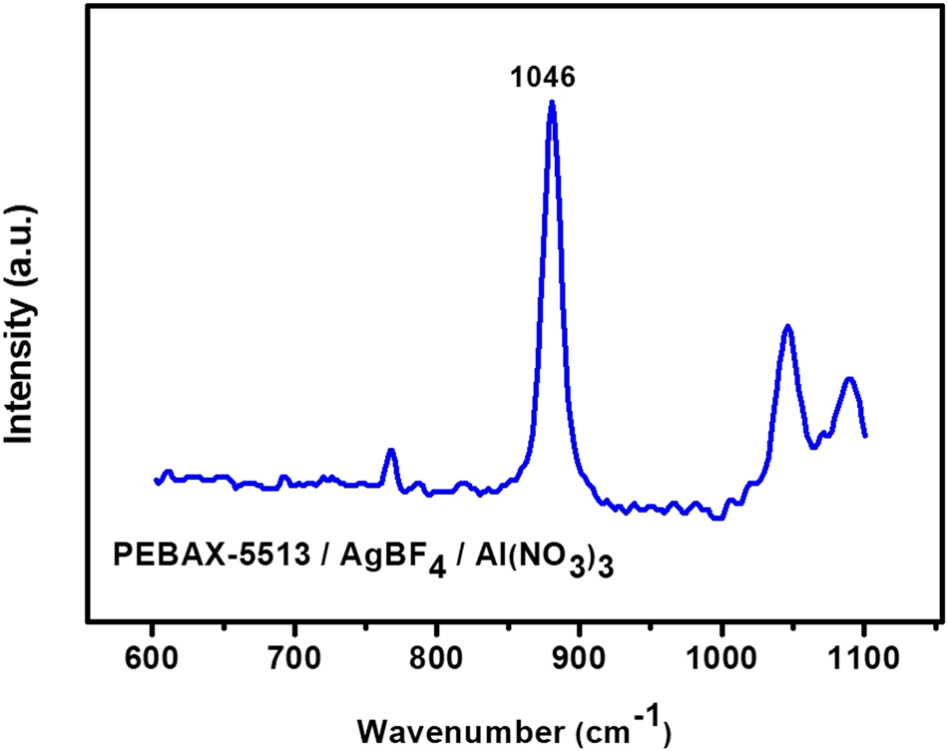
Raman spectra of NO_3_^−^ ions in PEBAX-5513/AgBF_4_/Al(NO_3_)_3._

Figures 5 and 6 showed the Raman graph used to confirm the state of the BF_4_^−^ and NO_3_^−^ ions. Figure 5 indicated the state of BF_4_^−^ ions, which meant free ions, ion pairs, and ion aggregates at 765, 770, and 774 cm^−1^, respectively. In the case of PEBAX-1657/AgBF_4_, BF_4_^−^ ions exist mainly in the form of free ions, but also in the form of ion pairs and ion aggregates^6^. On the other hand, in PEBAX-5513/AgBF_4,_ it was observed that BF_4_^−^ ions exists only as a free ion. This was due to the abundant amide groups in PEBAX-5513; therefore, the Ag^+^ ion could be strongly coordinated, being free of BF_4_^−^. In the case of PEBAX-1657/AgBF_4_/Al(NO_3_)_3_, the interaction between the Al^3+^ ions and BF_4_^−^ ions were generated. Then, BF_4_^−^ ions became the ion pairs state with Al^3+^ ions^6^. However, in PEBAX-5513/AgBF_4_/Al(NO_3_)_3_, the Al^3+^ ions was bound to the abundant amide; hence, BF_4_^−^ ions was expected to be able to maintain a free state. Figure 6 showed the state of the NO_3_^−^ ions, which indicated free ions, ion pairs, and ion aggregates, corresponding to 1034, 1040, and 1045 cm^−1^, respectively^23^. In PEBAX-5513/AgBF_4_/Al(NO_3_)_3_, the NO_3_^−^ ions react with Ag^+^ ions to form ion aggregates, corresponding to 1046 cm^−1^.

## Conclusions

**Figure 7:**
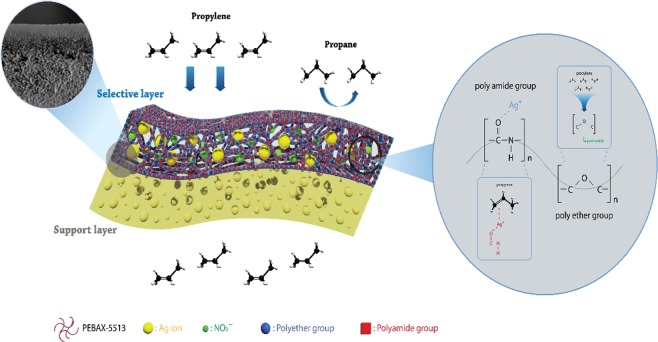
Facilitated propylene transport in PEBAX-5513/AgBF_4_/Al(NO_3_)_3._

The polymer matrix of PEBAX-1657/AgBF_4_/Al(NO_3_)_3_ used in an earlier study was changed to PEBAX-5513, with a higher amide group ratio in PEBAX, to improve the selectivity as shown in Fig. 7. The membrane permeance decreased because the proportion of the ether group, the permeable segment in PEBAX, decreased. However, there was no increase in selectivity. Since the ratio of amide was high, Ag^+^ ions were favorably in coordination with the oxygen of the carbonyl group, but the NO_3_^−^ ions had the enhanced interaction with Ag^+^ ions as obstacles for olefin complexation. As a result, the effect of the ratio of amide/ether in the polymer matrix could be negligible for Ag^+^ ions to act as olefin carriers for polymer/Ag salt/Al(NO_3_)_3_ complex membranes. Therefore, these results should be considered for the future design of block-copolymer syntheses to obtain high-selectivity/high-permeability membranes.

## Methods

### Materials

Poly(ether-block-amide)–5513 (PEBAX-5513) was manufactured by Arkema Inc. Silver tetrafluoroborate (AgBF_4_; 98%) was purchased from TCI Fine Chemicals. Aluminum nitrate nonahydrate (Al(NO_3_)_3_·9H_2_O; 98%) was purchased from Aldrich Co. All the chemicals were used as received.

### Preparation of membranes

PEBAX-5513 was dissolved in a mixed solvent of distilled water and ethanol at 90 °C to prepare a solution having a concentration of 3 wt%. The solution was allowed to cool at room temperature, and then AgBF_4_ was added as an olefin carrier so as to have a concentration of 17 wt% of the total solution. Al(NO_3_)_3_ was added as a reduction inhibitor of Ag^+^ ions so as to have a 0.1 mol ratio with respect to AgBF_4_. The solution was then stirred for 1 hour at room temperature and coated on the polysulfone support (Toray chemical, Inc., Korea) using an RK Control Coater (Model 202, Control Coater RK Print-Coat Instruments Ltd., UK). The solution-coated complex was dried in vacuum oven for 24 hours at room temperature.

### Gas separation experiments

The performance of PEBAX-5513/AgBF_4_/Al(NO_3_)_3_ membranes was tested using a propane/propylene mixed gas(50:50 vol%). The permeance of the membranes was obtained using a bubble flow meter and the selectivity was obtained using gas chromatography(GC). The flow of the mixed gas was controlled by a mass flow controller (MFC), and the gas permeance was expressed as GPU (1 GPU is 1 × 10^−6^ cm^3^ (STP)/(cm^2^ s cmHg)).

## Characterization

The prepared membranes were characterized by scanning electron microscopy (SEM; JSM-5600LV, JEOL). A VERTEX 70 FT-IR spectrometer (Bruker Optics Inc.) was used to investigate the chemical change in the membranes. The thermal stability of the membranes was measured by thermogravimetric analysis (TGA; Universal V4.5 A, TA Instruments). Raman spectra of the PEBAX-5513/AgBF_4_/Al(NO_3_)_3_ complex membrane were collected at room temperature using a Bruker Optics Ram II Raman module with a resolution of 4 cm^−1^.
